# Sub-Cellular Metabolomics Contributes Mitochondria-Specific Metabolic Insights to a Mouse Model of Leigh Syndrome

**DOI:** 10.3390/metabo11100658

**Published:** 2021-09-28

**Authors:** Gunter van der Walt, Jeremie Z. Lindeque, Shayne Mason, Roan Louw

**Affiliations:** Human Metabolomics, Faculty of Natural and Agricultural Sciences, North-West University, Potchefstroom 2531, South Africa; gvanderwalt007@gmail.com (G.v.d.W.); zander.lindeque@nwu.ac.za (J.Z.L.); nmr.nwu@gmail.com (S.M.)

**Keywords:** mitochondria, cytosol, mitochondrial disease, complex I deficiency, Ndufs4, metabolomics, sub-cellular metabolomics, ^1^H-NMR, LC-MS/MS

## Abstract

Direct injury of mitochondrial respiratory chain (RC) complex I by Ndufs4 subunit mutations results in complex I deficiency (CID) and a progressive encephalomyopathy, known as Leigh syndrome. While mitochondrial, cytosolic and multi-organelle pathways are known to be involved in the neuromuscular LS pathogenesis, compartment-specific metabolomics has, to date, not been applied to murine models of CID. We thus hypothesized that sub-cellular metabolomics would be able to contribute organelle-specific insights to known Ndufs4 metabolic perturbations. To that end, whole brains and skeletal muscle from late-stage Ndufs4 mice and age/sex-matched controls were harvested for mitochondrial and cytosolic isolation. Untargeted ^1^H-NMR and semi-targeted LC-MS/MS metabolomics was applied to the resulting cell fractions, whereafter important variables (VIPs) were selected by univariate statistics. A predominant increase in multiple targeted amino acids was observed in whole-brain samples, with a more prominent effect at the mitochondrial level. Similar pathways were implicated in the muscle tissue, showing a greater depletion of core metabolites with a compartment-specific distribution, however. The altered metabolites expectedly implicate altered redox homeostasis, alternate RC fueling, one-carbon metabolism, urea cycling and dysregulated proteostasis to different degrees in the analyzed tissues. A first application of EDTA-chelated magnesium and calcium measurement by NMR also revealed tissue- and compartment-specific alterations, implicating stress response-related calcium redistribution between neural cell compartments, as well as whole-cell muscle magnesium depletion. Altogether, these results confirm the ability of compartment-specific metabolomics to capture known alterations related to Ndufs4 KO and CID while proving its worth in elucidating metabolic compartmentalization in said pathways that went undetected in the diluted whole-cell samples previously studied.

## 1. Introduction

Throughout more than a century of mitochondrial research, science has ascertained at least this much regarding mitochondrial function: (i) it is critical to many facets of cellular health; (ii) it is a highly dynamic and adaptable role player in global and tissue-specific biology; and (iii) it maintains spatiotemporal compartmentalization of core metabolic/signaling networks. Mitochondria orchestrate these processes towards cellular homeostasis.

Through tightly regulated coordination with other intracellular spaces, mitochondria participate in several key energy-producing and biosynthetic pathways [1,2,3]. Furthermore, complex metabolic and signal transduction pathways integrate nutrient, stress state and cell cycle cues at the mitochondrial level to elicit whole-cell responses following any change in environment or physiological needs [4,5]. Concordantly, mitochondria have been implicated in multiple complex pathologies, from cancer to COVID-19 [6,7,8,9]. Central to our understanding of the mitochondrial contribution to pathology, however, is the study of primary mitochondrial disease (PMD), which results from deleterious mutations in core mitochondria-related genes [10,11]. Specifically, disorders of the first and foremost contributing enzyme of the respiratory chain (RC)—NADH:ubiquinone oxidoreductase (complex I, CI)—are the most prevalent, with multiple nDNA or mtDNA mutations contributing to the observed CI-deficient (CID) phenotypes [12,13]. Deletion of the iron–sulfur CI protein subunit, Ndufs4, leads to subsequent CID, and a progressive encephalomyopathy with severe musculoskeletal symptoms and poor prognosis known as Leigh syndrome (LS) [14,15]. The underlying whole-body knock-out (KO) paradoxically only leads to tissue-specific deterioration and metabolic reprogramming, implicating multiple biological systems in the observed mosaic.

Metabolomics has proven to be indispensable towards capturing changes in the small-molecule intermediates of metabolism that underlie bioenergetic, anabolic and signaling pathways intimately related to multiple mitochondria-related disease states [16,17,18]. Mouse models for PMD, such as the synthesized Ndufs4 KO strain studied here [19,20], serve as adequate in vivo models for CI-related LS and have contributed substantially to our understanding of the underlying tissue-specific deficits and potential treatment options [21,22]. In these mice, as in patients, it is known that whole-body Ndufs4 KO leads to graded, region-specific neurodegeneration and metabolic alteration, which has convincingly been shown to be the driving force of the total LS phenotype [23,24,25]. Oxidative and glycolytic muscles also show fiber type-specific metabolic alterations relating to the atrophy and locomotive defects in late-stage LS model mice and patients [26,27]. Both LS nervous and muscle tissues present features of perturbed metabolic and signaling pathways evident of differential catabolic/anabolic switching. These pathways are intertwined in the cascade of multi-organelle signaling molecules that facilitate an integrated stress response (ISR) during chronic mitochondrial stress [25].

Adaptive bioenergetic responses aimed at controlling the redox state while supplementing the tricarboxylic acid (TCA) cycle and RC-derived ATP synthesis are evident from the metabolite alterations in Ndufs4 brain and skeletal muscle tissues [24,26]. Features related to perturbed anabolic shifts are also prevalent, seemingly in line with the prerequisites of the ISR [22,28]. Concurrently, alterations in core amphibolic substrate levels—such as glutamine, glutamate and 2-ketoglutarate—are apparent even in lesser affected and pre-symptomatic neural/muscular Ndufs4 samples. Unlike in perturbed neural cells, which are supplemented by several neuron-glial metabolite shuttles [29,30], this anabolic shift is presumably far less sustainable in the comparably mitochondria-lacking glycolytic muscle tissues [31] and eventually leads to a global depletion of core metabolic intermediates. As such, induction of an amino acid restriction response has also been strongly correlated with RC inhibition in muscle fibers [32,33]. Evidently, multiple contributing and often conflicting metabolic switches are triggered during RC dysfunction, which require even deeper, more reductive and integrated approaches to fully grasp the metabolic dysfunction during Ndufs4 KO, LS and PMD overall.

Despite decades of study around the causes and effects of mitochondrial dysfunction, only recently has technology allowed us to observe the related metabolic effects inside mitochondria, specifically [34]. Mitochondria maintain a separate chemical milieu to the surrounding cytosol and organelles, with multiple unique, inter- and trans-compartmental reactions abundant. Mitochondria rely on tight membrane coupling to drive the proton gradient necessary for oxidative phosphorylation and ATP synthesis; thus, metabolite exchanges are highly coordinated by multiple substrate-specific, allosterically regulated, inner mitochondrial membrane transporter proteins- of which many are still being discovered and elucidated [35,36]. Finally, even in the most mitochondria-rich tissues, the total mitochondrial matrix volume is substantially smaller than the cytosolic space [37], leading to significant compartment-specific changes often being buried in the total cellular metabolome. This underlies the recent interest in metabolomics methods which can elucidate spatiotemporal compartmentalization of the implicated trans-cellular reactions and how this contributes to the observed whole-cell phenotype. Two complementary approaches underlie compartmental metabolomics studies. (1) Isotope-labeled metabolite flux measurements can follow the spatiotemporal distribution and the fate of certain targeted chemical precursors [38,39]. (2) Organelle isolation and metabolite fingerprinting offer a greater metabolic resolution and have concurrently delivered accurate sub-cellular insights on effects related to sex, age, fasting state, tumorigenesis and heavy metal toxicity [40,41,42,43]. While still scarce in volume, studies utilizing said methods in cell culture models of RC dysfunction have unequivocally proven mitochondria-level metabolite changes which cannot be deduced from whole-cell samples alone [39,44,45].

These techniques are still only finding their footing in the field of in vivo disease modeling at a handful of institutions [46] and, to the best of our knowledge, have yet to be applied to an in vivo model of genetic CID. We thus hypothesized that a rudimentary immunopurification-based organelle isolation and compartment-specific metabolomics strategy [47] could reveal mitochondria-specific metabolic insights that were not visible at the whole cell level. Comparative, multiplatform metabolomics was performed on the brain and skeletal muscle mitochondria and cytosol of late-stage Ndufs4 mice alongside wildtype (WT) control mice. The significantly altered features reported underline the current Ndufs4 metabolomics knowledge while indeed illuminating compartment-specific effects that went unseen in whole-cell studies of these tissues.

## 2. Results

### 2.1. Experimental Rationale and Data Quality

To elaborate on the current metabolic knowledge of Ndufs4-linked LS, we performed ^1^H-NMR and LC-MS/MS metabolomics on purified brain and quadriceps muscle mitochondria and cytosols from male Ndufs4 KO mice (*n* = 8), as well as age- and sex-matched controls (n = 8). The genotype was confirmed by an established PCR genotyping method. A priori sample calculations deemed these group sizes sufficient for detecting the most prominent metabolic effects in the current pilot (Cohen’s D ≥ 1.0 at 80% power; G*-Power 3 R-package [48]).

**Figure 1:**
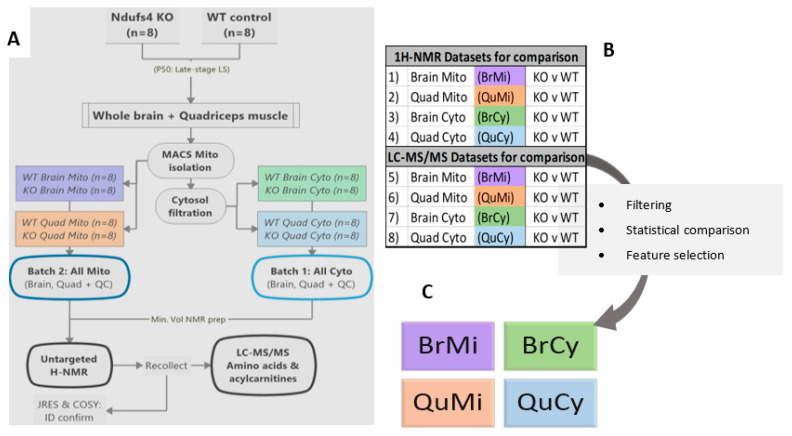
Summarized workflow for the study. Ndufs4 and control mouse quadriceps and whole brains were subjected to MACS mitochondrial isolation and cytosolic filtering, before subsequent ^1^H-NMR and LC-MS/MS analyses (**A**). Datasets were extracted as such that Ndufs4 KO and WT were compared in each matrix (**B**), before data processing and statistical feature selection. Thereafter, the combined significant feature (VIP) datasets for each tissue and compartment were further processed and interpreted (**C**).

An established adaptation of the ubiquitous Miltenyi MACS murine mitochondrial isolation kit for in vivo metabolomics was employed [47]—alongside cost-efficient, robust minimal-volume ^1^H-NMR and LC-MS/MS methods [26,49,50]—emphasizing the feasibility of mitochondrial metabolomics in more resource-restricted research settings. To maximize the information gained, post-NMR samples were subjected to LC-MS analyses, rather than splitting the already limited sample volume (Figure 1A). WT and KO means were compared in each sample matrix, whereafter the statistically significant metabolite alterations were interpreted across compartments for each tissue (Figure 1B,C). Absolute metabolite identity confirmation was provided by 2D-NMR techniques (JRES and COSY), as well as spectral matching to pure metabolite reference standards included for each mass transition probed by LC-MS/MS.

**Figure 2:**
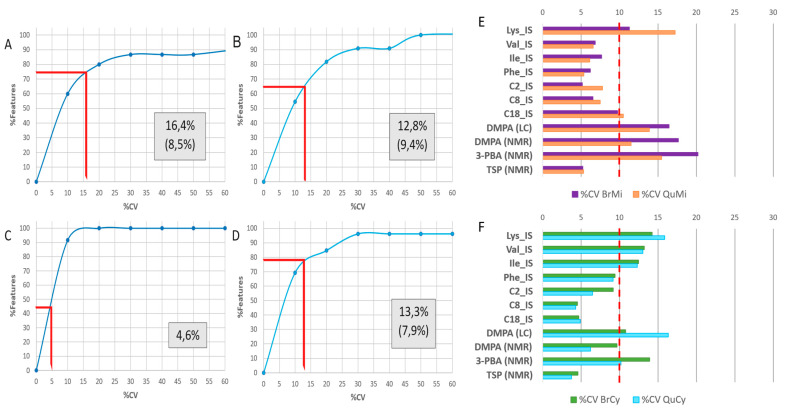
Technical variance and data quality for each ^1^H-NMR and LC-MS/MS batch. (**A**) Cumulative percentage features plotted over coefficient of variance (%CV) intervals—from QC sample technical replicates within ^1^H-NMR mitochondrial and (**B**) cytosolic sample batches. The same is plotted for (**C**) LC-MS/MS mitochondrial and (**D**) cytosolic batches as well. Enclosed numbers indicate mean %CV values across all viable features in each dataset—where individual unstable features were filtered, the number in brackets indicates the adjusted mean %CV after filtering. Measured IS compound variance as %CV in each tissue matrix, (**E**) for the mitochondrial and (**F**) cytosolic samples. Deuterium-labeled standards: C2_IS: d3-acetylcarnitine, C8_IS: d3-octanoylcarnitine, C18_IS: d3-stearoylcarnitine, Ile_IS: d10-isoleucine, Lys_IS: d4-lysine, Phe_IS: d5-phenylalanine, Val_IS: d3-valine. Other standards: DMPA: N,N-dimethylphenylalanine, 3-PBA: 3-phenylbutyric acid, TSP: 3-(trimethylsylil) propionic acid.

Data quality was controlled by monitoring univariate variance in quality control sample (QC) technical replicates throughout each batch as well as variance of IS compounds uniformly added to each sample. From the relative standard deviation, or percent coefficient of variance (%CV) reported for each compound (Figure 2A–D), the analytical accuracy of NMR or LC-MS measurements within these minimum-volume, buffered samples was well within acceptable precision limits for untargeted metabolomics [51]. Average QC-CV% values across all platforms were even below the FDA-stipulated 20% limit for targeted chemical analyses close to lower quantification limits [52], while no more than two compounds were removed from each dataset based on the selected 30% QC-CV filter employed here. From the NMR mitochondrial dataset, dimethylamine and dimethylformamide (possibly methylamine artefacts) were removed with a high %CV, while only alanine was excluded from analysis in the cytosolic NMR data. QC samples showed similar analytical performances in the LC-MS/MS batches, with only threonine being filtered from the cytosolic dataset, and none from the mitochondrial dataset. Upon exclusion of these four compounds, QC-CV values across all batches did not exceed 10%.

Finer technical variance, as inferred by IS compound %CVs across all samples per batch (Figure 2E,F), also averaged at ~10% for both cytosolic and mitochondrial batches, with no compound exceeding 20% CV. From the total signal scatterplots generated before and after data normalization (Figures S1 and S2), the sample-wise normalization strategy employed did not exacerbate the QC signal variance while also being able to correct for certain levels of technical variance in the separate sample groups analyzed in each batch. Therefore, it can be concluded that the metabolomics methods applied were of sufficient precision for metabolite abundance measurement while minimizing the inherent technical variance of low-volume mitochondrial metabolomics.

### 2.2. Metabolome Coverage and Data Clean-Up

**Figure 3:**
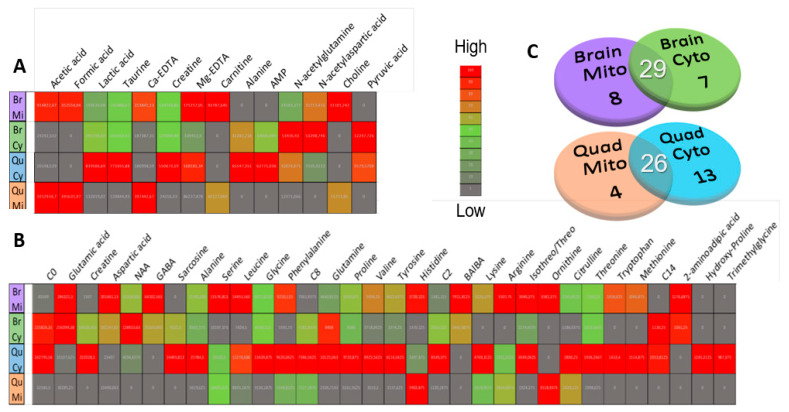
Comparison of viable metabolite presence and gross abundance across sample matrices. Venn diagrams in (**C**) show the number of metabolites viably detected above the noise level in mitochondria, cytosols and both compartments for the whole-brain and quadriceps matrices, separately. Heatmaps of average matrix raw peak abundances, contrasted by Euclidian between-mean distances, are presented for the (**B**) total viable LC-MS/MS and (**A**) ^1^H-NMR features. Br.C and Br.M abbreviate averages for whole-brain cytosolic and mitochondrial fractions, respectively, and Qu.C and Qu.M indicate quadriceps muscle cytosolic and mitochondrial groups.

Given current uncertainty regarding the exact mitochondrial metabolome and compartmentalization of ambiguous metabolite pools [34,44], we first established LC-MS/MS and ^1^H-NMR profiles for the accurately identified and quantifiable compound averages across each sample matrix. Untargeted ^1^H-NMR, following a binning approach, could identify and measure 14 compounds at various levels across all samples, while LC-MS/MS delivered 35 such viable compounds from the 54 metabolites originally targeted by MRM (Figure 3A,B). It should be noted that the LC-MS/MS methodology is biased towards amino acid and acylcarnitine analyses, targeting only mass transitions for a predefined set of metabolites from these classes.

From a tissue-specific perspective, whole-brain mitochondria and cytosols, respectively, delivered 8 and 7 compartment-specific compounds, and 29 across both compartments (Figure 3C). Similarly, 4 uniquely mitochondrial compounds were viably detected in the quadriceps, alongside 13 cytosolic and 26 cross-compartment metabolites. Detected and quantifiable features are listed according to total median spectral signal abundance and QC measurement stability in Table S1. The most abundant features across all matrices were glutamic acid, aspartic acid, carnitine, formic acid and lactic acid. Expectedly, several features were identified and analyzed in one compartment while not being present above baseline/detection levels in the other, as evident from the compartment-specific distributions of sarcosine, hydroxyproline, ornithine and formic acid, among others. This emphasizes the value of the reported approach in measuring metabolites at a mitochondrial level that are either ubiquitous in the total cell volume or diluted beyond detection limits therein.

Several MS feature peaks were removed due to being below the reliable signal/noise area range of 10^3^ mAu before the generation of the heatmaps shown in Figure 3. Thereafter, a 50% zero filter was employed, excluding compounds detected at < 1000 mAu in more than 8/16 samples. Finally, the 30% QC-CV data filter was applied as described in Section 2.1. These steps ensured only the most stable metabolites and most relevant patterns in the variation were preserved for statistical analyses.

### 2.3. Compartment-Specific, Significant Ndufs4 KO Metabolite Alterations

The selected variables of importance (VIPs) showing significant statistical (*p* < 0.1) and/or practical (*D* > 1.0) differences between WT and KO mitochondria and cytosols are reported in Table 1. The clean and normalized datasets were subjected to univariate tests for significant differences in means; *t*-test statistics and Cohen’s D values were calculated in SPSS, automatically accounting for formula derivations per feature based on their differential normality and homogeneity of variance criteria.

Significance limits stricter than the a priori power calculations infer, assure a greater preference for type II statistical errors (rejecting potentially significant changes) while minimizing type I errors (reporting non-significant changes as significant) [53,54]. Although this assures the few metabolites reported are indeed of significant value, many possibly significant alterations may be lost. Notably, three compounds approached the cut-off for significance in the whole-brain samples (*p* < 0.15; *D* > 0.8), while none but the reported quadriceps-derived metabolites showed borderline significant differences in means. Alanine, glutamine and sarcosine are thus included in the discussion of whole-brain metabolic perturbations. Altogether, these VIPs reverberate known bioenergetic and anabolic perturbations related to mitochondrial distress.

PLS-DA projections of the combined VIP datasets for each sample matrix indicate the power of the resulting model to separate WT and KO phenotypes based on the selected features from Table 1 (Figure S3). While the power of multivariate analyses is severely restricted by the small number of samples and significant features in each dataset, cross-validation of the PLS model indicates similar maximal model accuracies (~70%) across matrices. Despite the described limitations, these results also support the ability of sub-cellular metabolomics to discern between Ndufs4 and WT metabolic phenotypes.

### 2.4. Amino Acids Accumulate in Whole-Brain CI-Deficient Mitochondria to a Greater Extent than Cytosolic or Region-Specific Metabolomics Can Fully Elucidate

**Figure 4:**
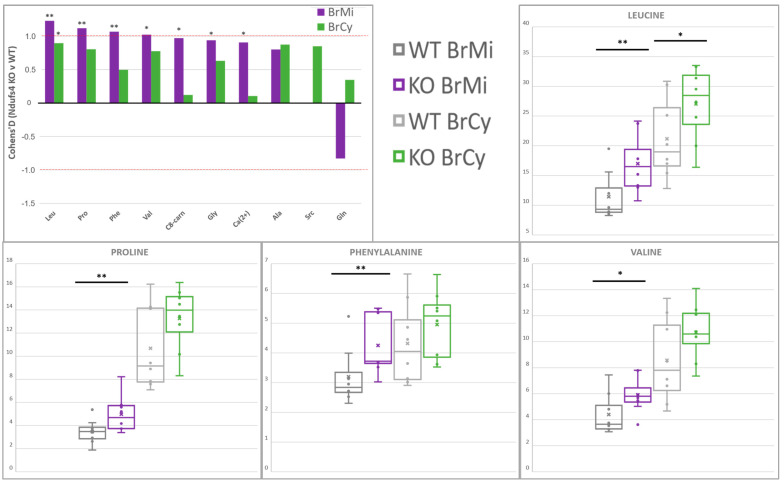
Compartment−specific metabolite alterations in Ndufs4 KO whole brain, identified by ^1^H−NMR and LC−MS/MS. Histograms indicate mean differences with size and directionality, graphed as Cohen’s D values, while boxplots summarize the sample value distributions for each compound across the two genotypes and cell compartments analyzed. Statistical significance via Student’s *t*-test is indicated as (*) for *p* < 0.1 or (**) for *p* < 0.05. Abbreviations: Ala: alanine, C8-carn: octanoylcarnitine, Ca(2+): Ca(II)-EDTA, Gln: glutamine, Gly: glycine, Leu: leucine, Phe: phenylalanine, Pro: proline, Src: sarcosine, Val: valine.

The majority of whole-brain compartment-specific metabolic alterations reported here (Table 1, Figure 4) pertain to metabolites at the intersections of bioenergetic and anabolic pathways. The overall increase in the branched-chain amino acids (BCAAs) valine (Val) and leucine (Leu) supports the classic model wherein hyperaccumulated NADH inhibits the NAD^+^-dependent enzyme branched-chain keto-acid dehydrogenase (BCKDH), leading to accumulation of its BCAA precursors [21,55,56]. These BCAA increases were more prominent at the mitochondrial level than previously detected in the lesser-affected anterior cortex, thus indicating a possibly predictive mitochondrial BCAA accumulation that whole-cell metabolic profiles cannot sufficiently convey [24]. A significant increase in proline (Pro) was seen only at the whole-brain mitochondrial level. This concurs Pro accumulation observed in all Ndufs4 brain regions [24,57], in line with the prominent role of mitochondrial–cytosolic Pro shuttling in buffering redox and adenylate charge ratios [58,59]. These results accentuate hypotheses regarding bioenergetic buffering during RC dysfunction while implicating spatial compartmentalization in these pathways.

Mitochondrial glycine (Gly) levels were significantly increased (Figure S4), while the same effect was not observed in the cytosolic samples or in whole-cell metabolite extracts from affected Ndufs4 brain regions [23,24]. As a key role player in several metabolic pathways in animal cells [60,61], multiple probable explanations exist which require further study. One such explanation involves one-carbon (1C) metabolism and its complex roles in mediating catabolic/anabolic shifts [62,63]. This notion is supported by an observed concomitant increase in cytosolic sarcosine (Src) levels, as an alternative 1C-related Gly precursor. Further amino acid increases appear obscure in isolation but, collectively, also support a general anabolic upregulation in these neural tissues [23]. The accumulation of alanine (Ala) across both compartments and increased mitochondrial phenylalanine (Phe) support a general increase in amino acid liberation for energy metabolism or protein synthesis [32,59], while all amino acid increases reported here may also support this hypothesis. Increased octanoylcarnitine (C8-carnitine) may correlate with a general dysregulation of fatty acid metabolism and neuroinflammation concurrent with the Ndufs4 phenotype [15,29], although these studies reported a greater dysregulation in acylcarnitines larger than C8-carnitine. In support of the glutamine (Gln) perturbation reported here, the prominent involvement of the Gln/Glu/ketoglutarate axis in the concurrent Ndufs4 brain’s metabolic shifts is well described, enacting substrate-level coordination of key amphibolic pathways [23,64]. Furthermore, some of these amino acid increases serve as potent stimulants of anabolic shifts, where Leu and Pro specifically upregulate the function of the mitochondrial target of rapamycin (mTORC1) signaling pathways towards greater protein and nucleic acid synthesis [65,66]. While sparsely spread across all these pathways, these results preliminarily reverberate perturbed anabolic signaling and metabolism, as hypothesized by current Ndufs4 brain studies [23,24,25].

Altogether, the isolated whole-brain mitochondria from Ndufs4 KO animals displayed patterns in metabolite perturbation that resemble the combined metabolic footprints of multiple brain regions previously measured in these mice while showing a greater perturbation at the mitochondria-specific level.

### 2.5. Differential Depletion of Quadriceps Mitochondrial and Cytosolic Metabolites Related to CID Muscle Metabolic Reprogramming

**Figure 5:**
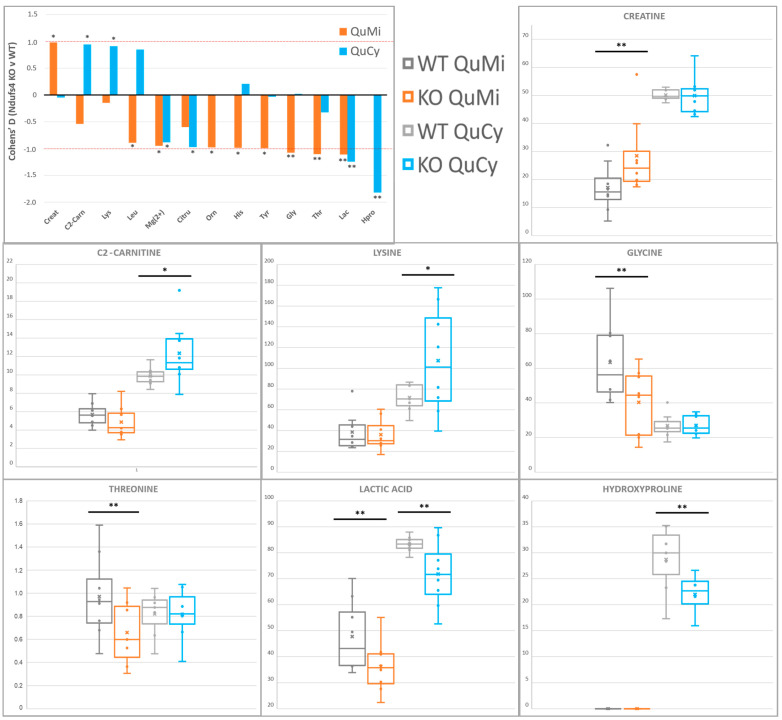
Compartment−specific metabolite alterations in Ndufs4 KO quadriceps muscle, identified by ^1^H−NMR and LC-MS/MS. Histograms indicate mean differences with size and directionality, graphed as Cohen’s D values, while boxplots summarize the sample value distributions for each compound across the two genotypes and cell compartments analyzed. Statistical significance via Student’s *t*-test is indicated as (*) for *p* < 0.1 or (**) for *p* < 0.05. Abbreviations: C2-carn: acetylcarnitine, Citru: citrulline, Creat: creatine, Gly: glycine, His: histidine, Hpro: 4-hydroxyproline, Lac: lactic acid, Leu: leucine, Lys: lysine, Mg(2+): Mg(II)-EDTA, Orn: ornithine, Src: sarcosine, Thr: threonine, Tyr: tyrosine.

Mitochondrial and cytosolic VIP compounds point to known perturbed bioenergetic pathways in Ndufs4 KO glycolytic skeletal muscle fibers (Table 1, Figure 5). A decrease in lactic acid (Lac) across both compartments was detected, in line with existing Ndufs4 whole-cell quadriceps metabolic data [26]. While sequestration of glycolytic units for NAD^+^ regeneration via mitochondrial glycerol-3-phosphate cycling stands as a convincing explanation for an Lac decrease at the whole cell level [59], concomitant mitochondria-level decreases also implicate biologically significant mitochondrial Lac import and oxidation towards TCA anaplerosis [67,68]. In that regard, a lesser-described mitochondrial Lac dehydrogenase isoform (m-LDH) seemingly plays an important role in skeletal muscle energy buffering during energetic stress [69]. An increase in acetylcarnitine (C2-carnitine), as reported here, is known to be a repercussion of acetyl-CoA accumulation due to RC blockage [70]. Accumulated mitochondrial creatine (Cr) contradicts the decrease in whole-cell Cr previously described in Ndufs4 KO quadriceps. This indicates perturbation of the mitochondrial–cytosolic creatine shuttle and differential functionalities of compartment-specific creatine kinase (CK) isoforms [71].

Significant depletion of select amino acids is also reminiscent of recent hypotheses supporting a cellular focus on alternate RC fueling pathways. Decreased 4-hydroxyproline (Hpro) indicates sequestration of upstream Pro for preferential use in balancing redox restrictions by proline/pyrroline-5-carboxylate (P5C) cycling, while it may also correlate with disturbed collagen proteostasis [57,72]. Increased cytosolic lysine (Lys) levels also indicate a shift in its interrelated metabolic pathways aimed at preserving cellular NADH/NAD^+^ ratios, effectively restricting its mitochondrial catabolism to 2-aminoadipate [26].

The measured ornithine (Orn) and citrulline (Citru) implicate compartmentalized perturbation of muscle urea cycling (Figure S5). While a decrease in these compounds supports a hypothesized increased channeling of Orn towards the Pro and/or CK shuttles as described above, distinct compartment-specific perturbations, as reported here, were not seen in a previous whole-cell Ndufs4 quadriceps study [26]. Gly was found significantly decreased in Ndufs4 muscle mitochondria while showing no significant difference in cytosolic samples. Threonine (Thr) levels followed the same trend, as Thr catabolizes through Gly back to Pyr—their concomitant depletions are likely related.

Decreased leucine (Leu), histidine (His) and tyrosine (Tyr) levels were also observed in the muscle mitochondria (Figure S5), indicative of perturbed amphibolic pathways. Other than being a critical energy source for the skeletal muscle, Leu also serves to increase whole-body energy availability by stimulating the muscle–liver Cahill cycle and subsequent hepatic gluconeogenesis [55,73], at the expense of muscle Lac and Glu. Classic His catabolism ultimately leads to replenishment of Glu and donation of methyl units to the 1C cycle; thus, its decrease may lie up- or downstream of RC-induced perturbations in these pathways [74]. While the role of lesser-expressed Tyr catabolic enzymes towards TCA anaplerosis is prominent in oncogenic muscle deterioration (cachexia) [75], significant perturbations in Tyr pools were not previously found at the cytosolic or whole cell level in MD mice [21,26].

Despite all the pathways described, the observed amino acid depletion is just as probably related to differential dysregulation of mitochondrial and cytosolic protein turnover during PMD [33,76]. Notably, this supports the hypothesis wherein the sparse, glycolytic muscle fiber mitochondrial network cannot sustain similar stress responses as induced in the brain and ultimately leads to an amino acid restriction response [31,32]. In summary, the described compartment-specific metabolite perturbations highlight several well-supported hypotheses regarding metabolic distress in CID glycolytic muscle fibers while clearly homing in on effects beyond the discernment ability of whole-cell metabolomics.

### 2.6. First Report of Tissue- and Compartment-Specific Bivalent Cation Measuremnt in Ndufs4 Mice Highlights Role of Metal Homeostasis in Integrated Stress Responses

**Figure 6:**
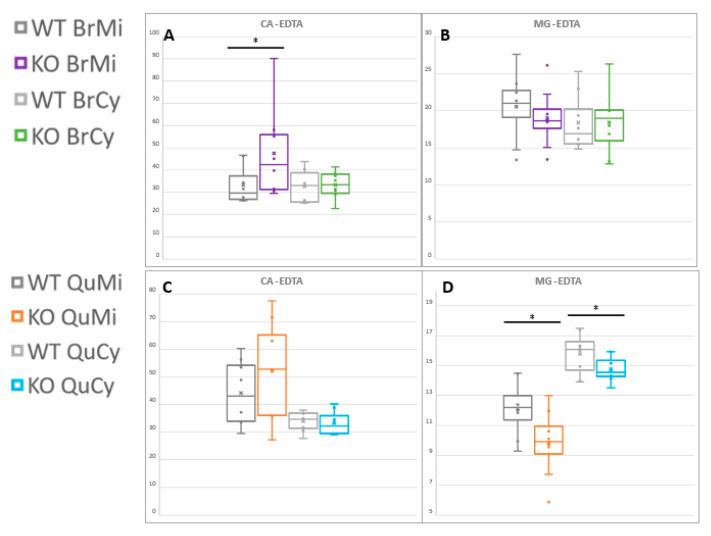
Compartment−specific magnesium and calcium levels, detected as EDTA chelates by ^1^H−NMR. (**A**) Brain mitochondrial Ca-EDTA and (**B**) Mg-EDTA, as well as (**C**) quadriceps muscle Ca-EDTA and (**D**) Mg-EDTA levels are shown. Statistical significance via Student’s *t*-test is indicated as (*) for *p* < 0.1. Ca-EDTA: Ca(II)-ethylenediaminetetraacetate, Mg-EDTA: Mg(II)-ethylenediaminetetraacetate.

During ^1^H-NMR analyses, we exploited a recently developed procedure for quantifying bivalent metal cations through their chelation with ethylenediaminetetraacetic acid (EDTA) [77,78]. As the sub-cellular samples contain a molar excess of EDTA as part of the isolation buffer environment [47,79], chelated calcium (Ca-EDTA) and magnesium (Mg-EDTA) concentrations are representative of the relative amounts of the cations present. Here, we report the first direct measurement (to our knowledge) of altered compartment-specific metallostasis in affected LS model tissues, as indicated in Figure 6.

A significant increase in brain mitochondrial Ca^2+^ levels was observed, with virtually no difference in its cytoplasmic concentrations due to Ndufs4 KO. This is in line with mitochondria–endoplasmic reticulum (ER) coupling and subsequent mitochondrial Ca^2+^ influx during ER stress responses to RC insult [1,25,80]. This buffering mechanism serves to upregulate multiple Ca^2+^-dependent metabolic enzymes, independently of cytosolic calcium pools. The skeletal muscle Mg^2+^ levels were significantly lower over both cell compartments. Although no present direct explanation suffices, lowered Mg^2+^ levels are also involved in bioenergetic feedback signaling during the mitochondrial stress responses [81,82]. This may also reflect alterations in protein turnover as described in the preceding section, as Mg^2+^ readily serves as a co-factor in multiple enzyme complexes, most notably in ATP-consuming proteins; however, whole-body Mg^2+^ decreases are often associated with decreased intestinal absorption and renal reabsorption due to energy deficiency [83].

## 3. Discussion

### 3.1. Contribution towards In Vivo Ndufs4 Model and In Vitro CID Knowledge Base

The reported mitochondrial and cytosolic metabolites from these LS model tissues highlight current metabolomic hypotheses underlying in vivo pathogenic and adaptive responses to Ndufs4 KO [15,21]. Central to the problem addressed by this study are detected metabolite alterations at the mitochondrial level in neural and glycolytic muscles that their correlated whole-cell and cytosolic samples do not reveal [23,24,26,84]. The whole-brain VIPs support the reported role of multiple bioenergetic and anabolic pathways at the core of the LS phenotype. In the related glycolytic muscle, differential patterns of metabolic change were observed across both compartments; however, all of them related to the presumed depletion of similar pathways to those upregulated in whole-brain tissues.

Both tissues clearly reverberate redox perturbations and the upregulation of pathways that attempt to restore reductant pools [59]. BCAA, Pro [56,72], Lac and Cr perturbations support this, with a greater visible effect in the mitochondrial samples. Most notably, the reported results indicate the roles of lesser-described mitochondrial mLDH and mCK isoforms, emphasizing the value of organelle-level resolution in these core classic bioenergetic pathways [67,71,85]. As mitochondrial NADH pools are presumably affected to a greater extent than the accompanying cytosolic pool [41,86], the greater observed perturbation of redox-related pathways at the sub-cellular level was expected. These results also further implicate core biosynthetic and anaplerotic pathways, as dysregulated anabolic signaling is a core feature and therapeutic target for CID [22,28]. Presumably, this shift attempts to replenish proteins that mediate cellular responses to energetic stress via mito-nuclear signaling and the ISR [4]. Specifically, altered levels of Leu, Pro, Gly and other 1C substrates underlie this statement [63,65,66], although the tight correlation between amino acid availability and protein synthesis may insinuate a direct correlation of all the reported amino acids with dysregulated protein turnover [76]. In contrast, increased liberation of free amino acids towards energy metabolism and concurrent RC/TCA congestion may also viably underlie the overall amino acid accumulation described here [26,62]. Regardless, mitochondrial proteostasis is regulated both separately and in unison with whole-cell levels [32]; therefore, the study of mitochondria-specific amino acid perturbations may well serve to deconvolute their differential metabolic fates under various conditions.

Very few studies to date have provided sub-cellular metabolomics data on RC distress; however, similar pathway perturbations and compartment-specific adaptations have been reported for in vitro CID models [39,44,45]. Ac-CoA and Lys accumulation, Glu/Gln disturbances and Pro and urea cycle upregulation have been reported in human and murine cells after CI inhibition by rotenone, but with cell type-specific patterns [44,45]. Our application of sub-cellular methods to a living mouse model thus also capitulates the value of extending these techniques to in vivo studies, by factoring in tissue-specific effects and considering systemic metabolic contributions. Whole-brain VIPs overall presented amphibolic pathway substrates that may drive the catabolic/anabolic shifts at the core of the phenotype [23]. In accordance, increased mitochondrial Ca^2+^ levels without a concomitant cytosolic increase indicate ER–mitochondrial contact—a key feature of ISR induction [80,87]. In vivo neural metabolomics, however, presents unique challenges regarding specialized, intercellular metabolic shuttles. For instance, astrocytic Leu serves as a key carbon source for neuronal TCA anaplerosis when this axis is depleted [88], while lactate and fatty acid shuttling between microglia and neurons is a key neuroinflammatory feature in late-stage LS [29,30]. Furthermore, the role of aromatic amino acids and Gly in neurotransmitter metabolism must be considered [23,89]. Glutamatergic neurons, however, are of predominant focus in LS models—implicating perturbed Gln/Glu/a-KG metabolism within their mitochondria [15,23,88].

The most striking contrast seen in the quadriceps tissue remains a global depletion of intermediates relating to the same pathways deemed increased in the Ndufs4 brain. Urea, Cr and Lac metabolic derangements were also unique to the muscle samples, in agreement with the current metabolic knowledge on mitochondria-related myopathies [26,33,90]. Lac and Leu depletions also implicate the muscle–liver Cahill cycle alongside the intracellular pathways described [55,73]. Of note, magnesium, and not calcium, was determined as the perturbed cation in this tissue. As a key co-factor for ATP-utilizing enzymes and an indicator of healthy protein turnover [81,82], this is a significant effect deserving of further study. Non-muscle metabolic contributions should, again, also be considered, as the Mg^2+^ depletion may also be related to reduced renal reabsorption or intestinal reabsorption due to energy deficits in these tissues under Ndufs4 KO [27,83].

In conclusion, rudimentary sub-cellular, mitochondria-targeted metabolomics captures known alterations in in vivo LS model mice while granting the ability to further elucidate minor tissue-specific and major mitochondrial responses to *Ndufs4* KO at a sub-cellular level. Herein lies the value of sub-cellular metabolomics for mitochondria-related research, as these effects were not detectable in whole-cell studies of the same tissues.

### 3.2. Limitations and Future Directions

Without the direct interrogation of several of the intermediates related to all the pathways discussed here, no mechanistic conclusions as to the full underlying significance of these effects can be established. In the face of many practical issues regarding technical variation in metabolomics studies using isolated mitochondria [34], the statistical selection criteria were set very strictly comparative to the statistical power-lacking small sample groups employed here. The purpose of this was to exclude the possibility of type I errors (false positives) while accepting the loss in information from an increased chance of type II error (falsely excluding positive correlations) [53,54] and thus focus on only the most prominent and abundant intergroup variation in this study.

Metabolic intermediates of significant interest but higher subjectivity to technical variance were thus not able to be quantified, while they are indirectly implicated in many of the implicated pathways. Concurrently, accurate analysis of compartmentalized Glu, Gln, Ser and Asp would deliver excellent insights on the LS pathology as these amino acids are critical intermediates to TCA anaplerosis, transamination reactions, urea cycling, 1C metabolism, biosynthetic pathways, cellular senescence and neurotransmitter metabolism. The quantification of 1C-related transsulfuration and methylation intermediates (Cys, cystathionine, methionine, etc.) would also serve to complement or rule out many of the hypothesized metabolic shifts described here. The methodological advancements published in the late course of this study allow even more targeted mitochondrial immunopurification techniques that promise interrogation of highly specified mitochondrial sub-populations from tissue matrices [46,91], while novel proteomics-based mitochondrial content assays may enable more accurate quantitative studies [92]. Regardless, the outcomes of this study accentuate the ability of sub-cellular metabolomics to further clarify the compartmentalized nature of pathways currently implicated in LS and other RC-related disease. Mitochondrial neuromyopathy research and clinical interventions thus stand to gain much from further targeted in vivo study of compartmentalized metabolic intermediate concentrations and fluxes for these anabolic and bioenergetic pathways.

## 4. Materials and Methods

### 4.1. Animals and Sampling

Male mice harboring a homozygous Ndufs4 truncation (KO) were used for this study, along with age- and sex-matched healthy controls (WT). Only males were included in this study to minimize known differences in sex-related metabolic variation which might occlude interpretation of the results [93,94]. KO mice were born from heterozygous crosses (Ndufs4^−/+^; B6.129S4-Ndufs4^tm1.1Rpa/J^) obtained from Jackson Laboratory (JAX stock #027058). Ndufs4 genotypes were confirmed by polymerase chain reaction using tail snips. The animals were bred and housed at the specific pathogen-free unit of the Vivarium (SAVC reg. no. FR15/13458) of the Pre-Clinical Drug Development Platform (PCDDP; NWU, RSA). This study was ethically approved (NWU-00568-19-A5) and governed by the Animal Research Ethics Committee of the NWU. Animals were housed under controlled conditions—temperature (22 ± 1 °C), humidity (55 ± 10%) and light (12:12 h light/dark cycle), with standard laboratory chow (Rodent Breeder, #RM1845, LabChef, Nutritionhub) and water provided ad libitum.

Based on survival curves and phenotypic analyses described in Miller et al. [95], mice of post-natal age of 45–50 days (P45–50) were selected for the study, as they phenotypically resemble the late stage of LS in patients. Mice were euthanized between P45 and 50 via cervical dislocation at the same time of day. Whole-brain and quadriceps femoris tissues were removed and rinsed with ice-cold saline solution (SABAX PBS; 0.9% NaCl (*w*/*v*), #7634, Adcock Ingram) to remove the surrounding blood and hair. These tissues were immediately processed for mitochondrial isolation.

### 4.2. Mitochondrial Isolation and Cytosol Preparation

To produce mitochondrial and cytosolic fractions of sufficient purity for metabolomics analysis, fractionated magnetic mitochondrial separation (FMSS) and cytosolic reverse filtration were performed according to a recently established kit-based method [47]. The process was performed at 4 °C using the MACS Mitochondria Extraction Kit for mouse tissue (Miltenyi Biotec) with slight variations from the manufacturer’s instructions [79]. Brains (100 mg) and quadriceps muscles (150 mg) were finely cut and homogenized in MACS lysis buffer to produce 10% and 15% (*w*/*v*) homogenates for the brains and muscles, respectively. Brief centrifugation at 1300× *g* (2 min) followed, before the post-nuclear supernatant was diluted 10× (*v*/*v*) in 1× separation buffer (SB).

For magnetic enrichment, mitochondria were labeled with anti-TOM22 microbeads (1.5 and 0.5 μL/mg tissue for brain and muscle samples, respectively) for 1 h at 4 °C. The suspension was loaded onto a magnetized LS column, in a MACS Separator at 4 °C to retain labeled mitochondria. Of the flow-through, regarded as a crude cytoplasm, 2.5 mL was subjected to centrifugal filtering to prepare the cytosolic fraction. Centrisart^®^ 100 kDa centrifugal cut-off filters (#13269E, Sigma Aldrich, St. Louis, MI, USA), accelerated to 2000× *g* for 15 min at 4 °C, were employed for the centrifugal filtering. Labeled mitochondria were eluted from the LS column in 1× SB after removal from the magnet—this was repeated 3× for each sample. Before further analysis, the eluates were centrifuged at 13,000× *g* for 2 min (4 °C), and the mitochondrial pellets were resuspended in 120 μL chromatography-grade water (Burdick & Jackson, Muskegon, MI, USA) containing the selected internal standard (IS) compounds described in Section 4.3.1. The mitochondria isolation procedure took approximately 90 min from tissue homogenization to mitochondrial elution.

### 4.3. Metabolomics Group Structure and Preparation for Multiplatform Studies

Established untargeted proton nuclear magnetic resonance (^1^H-NMR) and semi-targeted liquid chromatography-tandem mass spectrometry (LC-MS/MS) metabolomics methodologies [26,49] were applied to each mitochondrial and cytosolic sample produced from the quadriceps and whole-brain homogenates of each Ndufs4 KO (n = 8) or WT (n = 8) animal. To maximize the information gained from the small sample volumes available, samples were first analyzed with ^1^H-NMR, which requires minimal sample preparation, before preparing and analyzing the recollected samples on LC-MS/MS. All cytosolic samples were analyzed together as a batch on each platform, while the mitochondria were analyzed as their own separate batch on each platform.

#### 4.3.1. Internal Standards and Quality Control Samples

The IS compounds N,N-dimethyl-L-phenylalanine (DMPA; Sigma-Aldrich) and 3-phenylbutyric acid (3-PBA; Sigma-Aldrich) were added during the resuspension of the mitochondrial fractions as described above, while a small volume of the mixed stock solution thereof was added to each cytosolic fraction prior to metabolomics sample preparations to obtain similar concentrations of each IS across all samples. For each batch analyzed, a quality control (QC) sample (consisting of pooled aliquots from each sample therein) was periodically re-analyzed to monitor technical variation, while a single sample composed of pure chemical reference standards for each targeted LC-MS/MS feature was also analyzed at the end of each LC batch to aid metabolite identification. Unless otherwise stated, all solvents used were of chromatography grade or equivalent purity.

#### 4.3.2. Untargeted ^1^H-NMR Spectroscopy

Mitochondrial and cytosolic samples were prepared according to the recently established miniaturized ^1^H-NMR protocol [49], as follows: Samples were centrifugally filtered using Centrifree 3 kDa cut-off filters (Merck/Millipore) at 12,000× *g* and room temperature to remove proteins and other macromolecules, before 54 μL filtrate was transferred to a 2 mm NMR tube using the eVol^®^ NMR digital syringe (Sigma-Aldrich). The programmed pipetting sequence added the selected chemical shift standard for ^1^H-NMR (3-(trimethylsylil)-propionic acid (TSP; Merck/MagniSolv™) in deuterated water (D_2_O)), such that a 10:90% ratio of D_2_O/H_2_O was maintained as per the standard protocol. The 2 mm NMR tube was inserted into the gripper adaptor of the Bruker MATCH system, before each NMR MATCH assembly was loaded onto a SampleXpress autosampler for NMR analysis.

Analysis proceeded at 500 MHz on a Bruker Avance III HD NMR system, equipped with a 5 mm TXI probe head optimized for ^1^H observation. Briefly, ^1^H spectra were acquired as 128 transients in 32 K data points with a spectral width of 6000 Hz (12.0 ppm). The H_2_O resonance at 4.70 ppm was suppressed using the pulse sequence program NOESY-presat. With the number of dummy scans = 4 and the number of scans = 128, this yielded a run time of 15.75 min per sample. Each sample was automatically shimmed on the deuterium signal, locked, probe tuned and matched and pulse calibrated using Bruker Topspin (V3.5), before further processing was conducted using Bruker AMIX (V3.9.14).

NMR samples were recollected post-analyses for LC-MS/MS preparation, as the employed NMR methodology employed is innately non-destructive. Of the recollected samples, 41 µL of each was transferred to separate microcentrifuge tubes for LC-MS analyses, while the approximately 19 µL remainder was pooled for each tissue matrix (brain and quad cyto, brain and quad mito) to perform additional 2D correlation spectroscopy (COSY) and 2D J-resolved spectroscopy (JRES) to confirm metabolite identification [96].

#### 4.3.3. LC-MS/MS Amino Acid and Acylcarnitine Profiling

LC-MS/MS amino acid and acylcarnitine profiling was performed according to the methodology previously described in [26] for murine model tissue samples—with minor modifications to better suit the post-MACS mitochondrial and cytosolic sample matrices.

To each post-NMR extract (41 µL), an internal standard mixture consisting of several deuterium-labeled versions of selected target compounds (2.5 ppm per compound) was added before LC-MS/MS sample preparation (valine_d8, isoleucine_d10, phenylalanine_d5, lysine_d4, acetylcarnitine_d3, octanoylcarnitine_d3, octadecanoylcarnitine_d3; all from Cambridge Isotope Laboratories). Metabolites were extracted in a molar excess (~300 µL) of acetonitrile at 4 °C (10 min) before 10 min centrifugation (20,000× *g* at 4 °C). The metabolite-containing supernatant was thereafter evaporated under nitrogen gas stream. Metabolites were butylated via a reaction with 4:1 (*v*/*v*) 1-butanol (Sigma-Aldrich)/acetyl chloride (Fluka Analytical) and heatblock incubation at 50 °C for 1 h. Samples were again evaporated under nitrogen stream, before being reconstituted in a final volume of 100 µL water/acetonitrile (50:50, *v*/*v*; Burdick & Jackson) and transferred to 250 μL pulled point glass vial inserts.

The LC-MS/MS system consisted of an Agilent© 1200 series HPLC front end, coupled to an Agilent© 6410 series triple quadrupole mass analyzer with an electrospray ionization (ESI) source, operated in the positive ionization mode. Analytes were separated on a C18 Zorbax SB-Aq reverse phase column (Agilent©, 2.1 mm × 150 mm × 3.5 μm) kept at 30 °C. A sample volume of 2.5 µL was separated using a mobile phase gradient. The chromatographic gradient started at 100% solvent A (water with 0.1% formic acid (Sigma-Aldrich)) and 0% solvent B (acetonitrile with 0.1% formic acid), with a flow of 0.3 mL/min, maintained for 1 min, before ramping to 5% solvent B over 1 min. Thereafter, solvent B was increased to 20% over a period of 2 min, where it was then kept constant for 3 min. Thereafter, it was increased linearly to 100% solvent B over 7 min. Over this period, the flow was linearly increased to 0.35 mL/min (between 14 and 14.1 min). After maintaining these conditions for 5 min, the gradient was decreased to 5% solvent B at 19.5 min and kept constant for 1.5 min. A post-run of 8 min was allowed to ensure equilibration of the column between samples, capping the 29 min total run time. The ESI source gas (nitrogen) temperature was kept at 300 °C, with a flow rate of 7.5 L/min. Nebulizer pressure was kept at 30 psi, and the capillary voltage at 3500 V. The Agilent© MassHunter Workstation Software (v B09.00) was used for data acquisition and extraction.

#### 4.3.4. Data Processing

Spectral data were extracted into matrices, where an extra NMR data matrix reporting only the untargeted spectral bin sums for each sample was also extracted for calculation of a total useful NMR signal (NMR-TUS) normalization factor, as none of the selected IS compounds could accurately represent the variance due to pre-metabolomics tissue sampling and MACS isolation procedures. All datasets were individually inspected for data quality, through correct peak picking, alignment and batch precision (Figure 2) as IS and QC coefficients of variance (CV%), and preprocessed (50% QC-CV% filtering, missing value imputation via randomized ¼ minimum area replacement and normalization).

Metabolites were normalized to tissue mass through normalization with NMR-TUS as the most applicable factor for the data reported here (Figures S2 and S3). For LC-MS/MS data, metabolites were normalized to their own isotope (where possible) or the isotope showing the strongest linear correlation thereto, based on the analyte peak area (Pearson’s r correlation analyses) prior to the above-mentioned normalizations. No significant batch effects were evident; thus, no batch corrections were performed.

Finally, data pretreatment (glog transformation and Pareto scaling) and outlier detection (PCA Hotelling’s T2 ellipses, loadings plot and biplots) were conducted. Data processing was conducted using Excel 2013 (Microsoft), SPSS Statistics (v.25.0; IBM) and MetaboAnalyst 5.0 [97]. The metabolomics data obtained in this study (file name: Gunter007dekok_20210916_012612) can be accessed at the Common Fund’s National Metabolomics Data Repository (NMDR) website, the Metabolomics Workbench (https://www.metabolomicsworkbench.org (accessed on 18 September 2021), where it has been assigned the Data Track ID 2843).

#### 4.3.5. Statistical Analyses

Univariate analyses were used to determine significant discriminatory features between Ndufs4 KO and WT sub-cellular metabolic profiles. Comparisons were performed between genotypes (WT vs. KO) in each separate tissue compartment dataset only, with only the results of these tests being compared between compartments and tissues.

An independent, two-sample Student *t*-test was carried out on the unpretreated, NMR-TUS-normalized datasets to identify compounds with significantly altered intensities between diseased mice and controls (VIPs). In line with power calculations performed (G* Power 3; Universität Dusseldorf) at the inception of this study, a t-statistic with a *p*-value of <0.1 was considered statistically significant, while effect sizes (Cohen’s D value; D) of ≥1.0 were considered practically significant and used complimentary to the *t*-test [48]. Principal component analysis (PCA) and partial least squares discriminant analyses (PLS-DA) were used to visually investigate the clustering, covariance and discriminatory power of the univariately selected VIPs. This was performed in MetaboAnalyst 5.0 [97] on a log-transformed and Pareto-scaled dataset comprising only the important metabolites identified on each platform.

Dataset features were identified via spectral and retention time matching using the analyzed pure chemical reference standards, and commercial and in-house spectral libraries together with public databases and metabolite identities were assigned to spectral features, all with the highest (Level 1) confidence [39,40].

## Figures and Tables

**Table 1:** VIP compounds identified by univariate statistical and practical significance testing. Common metabolite names (Level 1 identity) are reported with the direction of means change (**↑** and **↓** arrows representing KO vs WT mean increases or decreases, respectively). *T*-test *p*-values and Cohen’s D values in the cytosol—alongside the correlating mitochondrial KO vs. WT comparison are shown in each tissue. The metabolomics platform from which each significant feature was derived is also shown. N.S.: non-significant statistical test result, N.Q.: not quantified due to being filtered from dataset, N.D.: not detected above baseline level.

Quadriceps Muscle							
*Cytosol*				*Mitochondria*			
Metabolite (Level 1)	KO ∆ (vs. WT)	*p*	*D*	KO ∆ (vs. WT)	*p*	*D*	Platform
Lactic acid	↓	0.04	1.2	↓	0.04	1.1	^1^H-NMR
Mg(II)-EDTA	↓	0.10	0.9	↓	0.09	0.9	^1^H-NMR
Threonine	N.Q.			↓	0.04	1.1	LC-MS/MS
Glycine	N.S.			↓	0.05	1.1	LC-MS/MS
Creatine	N.S.			↑	0.05	1.0	^1^H-NMR
Histidine	N.S.			↓	0.07	1.0	LC-MS/MS
Tyrosine	N.S.			↓	0.07	1.0	LC-MS/MS
Ornithine	N.D.			↓	0.08	1.0	LC-MS/MS
Leucine	N.S.			↓	0.10	0.9	LC-MS/MS
Hydroxyproline	↓	0.01	1.8	N.Q.			LC-MS/MS
Citrulline	↓	0.07	1.0	N.S.			LC-MS/MS
Acetylcarnitine	↑	0.09	0.9	N.S.			LC-MS/MS
Lysine	↑	0.10	0.9	N.S.			LC-MS/MS
**Whole Brain**							
***Cytosol***				***Mitochondria***			
**Metabolite** **(Level 1)**	**KO ∆** **(vs. WT)**	***p***	***D***	**KO ∆** **(vs. WT)**	***p***	***D***	**Platform**
Leucine	↑	0.09	0.9	↑	0.03	1.2	LC-MS/MS
Proline	N.S.			↑	0.03	1.1	LC-MS/MS
Phenylalanine	N.S.			↑	0.03	1.1	LC-MS/MS
Valine	N.S.			↑	0.06	1.0	LC-MS/MS
Octanoylcarnitine	N.S.			↑	0.08	1.0	LC-MS/MS
Glycine	N.S.			↑	0.08	0.9	LC-MS/MS
Ca(II)-EDTA	N.S.			↑	0.09	0.9	^1^H-NMR
Alanine ^1^	↑	0.10	0.9	↑	0.13	0.8	LC-MS/MS
Glutamine ^1^	N.S.			↓	0.12	0.8	LC-MS/MS
Sarcosine ^1^	↑	0.11	0.8	N.D.			LC-MS/MS

^1^ Metabolites approaching statistical and practical significance, yet below defined limits.

## Data Availability

The metabolomics data obtained in this study can be accessed at the Common Fund’s National Metabolomics Data Repository (NMDR) website, the Metabolomics Workbench (https://www.metabolomicsworkbench.org (accessed on 18 September 2021), where it has been assigned the Data track ID: 2843, and the file name Gunter007dekok_20210916_012612).

## Supplementary Materials

The following are available online at https://www.mdpi.com/article/10.3390/metabo11100658/s1, Table S1: Positively detected features and measurement stability, Figures S1 and S2: Comparison of selected data normalization factors for LC-MS/MS and NMR batches, respectively, Figure S3: PLS-DA group separation based on selected VIPs, Figures S4 and S5: Additional VIP feature boxplots for brain and quadriceps groups, respectively.

## References

[1] Nunnari J., Suomalainen A. (2012). Mitochondria: In sickness and in health. Cell.

[2] Shaughnessy D.T., McAllister K., Worth L., Haugen A.C., Meyer J.N., Domann F.E., Houten B.V., Mostoslavsky R., Bultman S.J., Baccarelli A.A. (2014). Mitochondria, Energetics, Epigenetics, and Cellular Responses to Stress. Environ. Health Perspect..

[3] Aouacheria A., Baghdiguian S., Lamb H.M., Huska J.D., Pineda F.J., Hardwick J.M. (2017). Connecting mitochondrial dynamics and life-or-death events via Bcl-2 family proteins. Neurochem. Int..

[4] Quirós P.M., Mottis A., Auwerx J. (2016). Mitonuclear communication in homeostasis and stress. Nat. Rev. Mol. Cell Biol..

[5] Wasilewski M., Chojnacka K., Chacinska A. (2017). Protein trafficking at the crossroads to mitochondria. Biochim. Biophys. Acta Mol. Cell Res.

[6] Eysert F., Kinoshita P.F., Mary A., Vaillant-Beuchot L., Checler F., Chami M. (2020). Molecular Dysfunctions of Mitochondria-Associated Membranes (MAMs) in Alzheimer’s Disease. Int. J. Mol. Sci..

[7] Wallace D.C. (2012). Mitochondria and cancer. Nat. Rev. Cancer.

[8] Saleh J., Peyssonnaux C., Singh K.K., Edeas M. (2020). Mitochondria and microbiota dysfunction in COVID-19 pathogenesis. Mitochondrion.

[9] Stepien K.M., Heaton R., Rankin S., Murphy A., Bentley J., Sexton D., Hargreaves I.P. (2017). Evidence of Oxidative Stress and Secondary Mitochondrial Dysfunction in Metabolic and Non-Metabolic Disorders. J. Clin. Med..

[10] Ylikallio E., Suomalainen A. (2012). Mechanisms of mitochondrial diseases. Ann. Med..

[11] Craven L., Alston C.L., Taylor R.W., Turnbull D.M. (2017). Recent advances in mitochondrial disease. Annu. Rev. Genom. Hum. Genet..

[12] Triepels R., Van Den Heuvel L., Trijbels J., Smeitink J. (2001). Respiratory chain complex I deficiency. Am. J. Med. Genet..

[13] Ghezzi D., Zeviani M. (2018). Human diseases associated with defects in assembly of OXPHOS complexes. Essays Biochem..

[14] Lake N.J., Bird M.J., Isohanni P., Paetau A. (2015). Leigh syndrome: Neuropathology and pathogenesis. J. Neuropathol. Exp. Neurol..

[15] Quintana A., Kruse S.E., Kapur R.P., Sanz E., Palmiter R.D. (2010). Complex I deficiency due to loss of Ndufs4 in the brain results in progressive encephalopathy resembling Leigh syndrome. Proc. Natl. Acad. Sci. USA.

[16] Newgard C.B. (2017). Metabolomics and Metabolic Diseases: Where Do We Stand?. Cell Metab..

[17] Priori R., Scrivo R., Brandt J., Valerio M., Casadei L., Valesini G., Manetti C. (2013). Metabolomics in rheumatic diseases: The potential of an emerging methodology for improved patient diagnosis, prognosis, and treatment efficacy. Autoimmun. Rev..

[18] Nagrath D., Caneba C., Karedath T., Bellance N. (2011). Metabolomics for mitochondrial and cancer studies. Biochim. Biophys. Acta.

[19] Kruse S.E., Watt W.C., Marcinek D.J., Kapur R.P., Schenkman K.A., Palmiter R.D. (2008). Mice with mitochondrial complex I deficiency develop a fatal encephalomyopathy. Cell Metab..

[20] Leong D.W., Komen J.C., Hewitt C.A., Arnaud E., McKenzie M., Phipson B., Bahlo M., Laskowski A., Kinkel S.A., Davey G.M. (2012). Proteomic and metabolomic analyses of mitochondrial complex I-deficient mouse model generated by spontaneous B2 short interspersed nuclear element (SINE) insertion into NADH dehydrogenase (ubiquinone) Fe-S protein 4 (Ndufs4) gene. J. Biol. Chem..

[21] Esterhuizen K., van der Westhuizen F.H., Louw R. (2017). Metabolomics of mitochondrial disease. Mitochondrion.

[22] Johnson S.C., Yanos M.E., Kayser E.B., Quintana A., Sangesland M., Castanza A., Uhde L., Hui J., Wall V.Z., Gagnidze A. (2013). mTOR inhibition alleviates mitochondrial disease in a mouse model of Leigh syndrome. Science.

[23] Johnson S.C., Kayser E.B., Bornstein R., Stokes J., Bitto A., Park K.Y., Pan A., Sun G., Raftery D., Kaeberlein M. (2020). Regional metabolic signatures in the Ndufs4(KO) mouse brain implicate defective glutamate/alpha-ketoglutarate metabolism in mitochondrial disease. Mol. Genet. Metab..

[24] Terburgh K., Coetzer J., Lindeque J.Z., van der Westhuizen F.H., Louw R. (2021). Aberrant BCAA and glutamate metabolism linked to regional neurodegeneration in a mouse model of Leigh syndrome. Biochim. Biophys. Acta Mol. Basis Dis..

[25] Emmerzaal T.L., Preston G., Geenen B., Verweij V., Wiesmann M., Vasileiou E., Gruter F., de Groot C., Schoorl J., de Veer R. (2020). Impaired mitochondrial complex I function as a candidate driver in the biological stress response and a concomitant stress-induced brain metabolic reprogramming in male mice. Transl. Psychiatry.

[26] Terburgh K., Lindeque Z., Mason S., van der Westhuizen F., Louw R. (2019). Metabolomics of *Ndufs4*^−/−^ skeletal muscle: Adaptive mechanisms converge at the ubiquinone-cycle. Biochim. Biophys. Acta Mol. Basis Dis..

[27] Finsterer J. (2008). Leigh and Leigh-like syndrome in children and adults. Pediatr. Neurol..

[28] Khan N.A., Nikkanen J., Yatsuga S., Jackson C., Wang L., Pradhan S., Kivela R., Pessia A., Velagapudi V., Suomalainen A. (2017). mTORC1 Regulates Mitochondrial Integrated Stress Response and Mitochondrial Myopathy Progression. Cell Metab..

[29] Liu L., MacKenzie K.R., Putluri N., Maletić-Savatić M., Bellen H.J. (2017). The Glia-Neuron Lactate Shuttle and Elevated ROS Promote Lipid Synthesis in Neurons and Lipid Droplet Accumulation in Glia via APOE/D. Cell Metab..

[30] Mason S. (2017). Lactate Shuttles in Neuroenergetics—Homeostasis, Allostasis and Beyond. Front. Neurosci..

[31] Schiaffino S., Reggiani C. (2011). Fiber types in mammalian skeletal muscles. Physiol. Rev..

[32] Johnson M.A., Vidoni S., Durigon R., Pearce S.F., Rorbach J., He J., Brea-Calvo G., Minczuk M., Reyes A., Holt I.J. (2014). Amino acid starvation has opposite effects on mitochondrial and cytosolic protein synthesis. PLoS ONE.

[33] Tyynismaa H., Carroll C.J., Raimundo N., Ahola-Erkkila S., Wenz T., Ruhanen H., Guse K., Hemminki A., Peltola-Mjosund K.E., Tulkki V. (2010). Mitochondrial myopathy induces a starvation-like response. Hum. Mol. Genet..

[34] Liu X., Xu G. (2018). Recent advances in using mass spectrometry for mitochondrial metabolomics and lipidomics—A review. Anal. Chim. Acta.

[35] Palmieri F. (2013). The mitochondrial transporter family SLC25: Identification, properties and physiopathology. Mol. Asp. Med..

[36] Hewton K.G., Johal A.S., Parker S.J. (2021). Transporters at the Interface between Cytosolic and Mitochondrial Amino Acid Metabolism. Metabolites.

[37] Kaasik A., Safiulina D., Zharkovsky A., Veksler V. (2007). Regulation of mitochondrial matrix volume. Am. J. Physiol. Cell Physiol..

[38] Wiechert W. (2001). 13C metabolic flux analysis. Metab. Eng..

[39] Gravel S.P., Andrzejewski S., Avizonis D., St-Pierre J. (2014). Stable isotope tracer analysis in isolated mitochondria from mammalian systems. Metabolites.

[40] Roede J.R., Park Y., Li S., Strobel F.H., Jones D.P. (2012). Detailed mitochondrial phenotyping by high resolution metabolomics. PLoS ONE.

[41] Goodman R.P., Calvo S.E., Mootha V.K. (2018). Spatiotemporal compartmentalization of hepatic NADH and NADPH metabolism. J. Biol. Chem..

[42] Go Y.M., Roede J.R., Orr M., Liang Y., Jones D.P. (2014). Integrated redox proteomics and metabolomics of mitochondria to identify mechanisms of cd toxicity. Toxicol. Sci..

[43] Kiebish M.A., Han X., Cheng H., Seyfried T.N. (2009). In vitro growth environment produces lipidomic and electron transport chain abnormalities in mitochondria from non-tumorigenic astrocytes and brain tumours. ASN Neuro.

[44] Chen W.W., Freinkman E., Wang T., Birsoy K., Sabatini D.M. (2016). Absolute Quantification of Matrix Metabolites Reveals the Dynamics of Mitochondrial Metabolism. Cell.

[45] Pan D., Lindau C., Lagies S., Wiedemann N., Kammerer B. (2018). Metabolic profiling of isolated mitochondria and cytoplasm reveals compartment-specific metabolic responses. Metabolomics.

[46] Bayraktar E.C., Baudrier L., Ozerdem C., Lewis C.A., Chan S.H., Kunchok T., Abu-Remaileh M., Cangelosi A.L., Sabatini D.M., Birsoy K. (2019). MITO-Tag Mice enable rapid isolation and multimodal profiling of mitochondria from specific cell types in vivo. Proc. Natl. Acad. Sci. USA.

[47] van der Walt G., Louw R. (2020). Novel mitochondrial and cytosolic purification pipeline for compartment-specific metabolomics in mammalian disease model tissues. Metabolomics.

[48] Faul F., Erdfelder E., Lang A.-G., Buchner A. (2007). G* Power 3: A flexible statistical power analysis program for the social, behavioral, and biomedical sciences. Behav. Res. Methods.

[49] Mason S., Terburgh K., Louw R. (2018). Miniaturized ^1^H-NMR method for analyzing limited-quantity samples applied to a mouse model of Leigh disease. Metabolomics.

[50] Mels C., Jansen van Rensburg P., van der Westhuizen F.H., Pretorius P.J., Erasmus E. (2011). Increased excretion of C4-carnitine species after a therapeutic acetylsalicylic acid dose: Evidence for an inhibitory effect on short-chain fatty acid metabolism. Int. Sch. Res. Not..

[51] Dunn W.B., Wilson I.D., Nicholls A.W., Broadhurst D. (2012). The importance of experimental design and QC samples in large-scale and MS-driven untargeted metabolomic studies of humans. Bioanalysis.

[52] US FDA (2001). Guidance for Industry: Bioanalytical Method Validation.

[53] Vinaixa M., Samino S., Saez I., Duran J., Guinovart J.J., Yanes O. (2012). A Guideline to Univariate Statistical Analysis for LC/MS-Based Untargeted Metabolomics-Derived Data. Metabolites.

[54] Akobeng A.K. (2016). Understanding type I and type II errors, statistical power and sample size. Acta Paediatr..

[55] Bifari F., Nisoli E. (2017). Branched-chain amino acids differently modulate catabolic and anabolic states in mammals: A pharmacological point of view. Br. J. Pharmacol..

[56] Arany Z., Neinast M. (2018). Branched Chain Amino Acids in Metabolic Disease. Curr. Diabetes Rep..

[57] Oka K., Ohya-Shimada W., Mizuno S., Nakamura T. (2013). Up-regulation of cyclin-E1 via proline-mTOR pathway is responsible for HGF-mediated G1/S progression in the primary culture of rat hepatocytes. Biochem. Biophys. Res. Commun..

[58] Krishnan N., Dickman M.B., Becker D.F. (2008). Proline modulates the intracellular redox environment and protects mammalian cells against oxidative stress. Free Radic. Biol. Med..

[59] McDonald A.E., Pichaud N., Darveau C.A. (2018). “Alternative” fuels contributing to mitochondrial electron transport: Importance of non-classical pathways in the diversity of animal metabolism. Comp. Biochem. Physiol. B Biochem. Mol. Biol..

[60] Razak M.A., Begum P.S., Viswanath B., Rajagopal S. (2017). Multifarious Beneficial Effect of Nonessential Amino Acid, Glycine: A Review. Oxidative Med. Cell. Longev..

[61] Wang W., Wu Z., Dai Z., Yang Y., Wang J., Wu G. (2013). Glycine metabolism in animals and humans: Implications for nutrition and health. Amino Acids.

[62] Yang L., Garcia Canaveras J.C., Chen Z., Wang L., Liang L., Jang C., Mayr J.A., Zhang Z., Ghergurovich J.M., Zhan L. (2020). Serine Catabolism Feeds NADH when Respiration Is Impaired. Cell Metab..

[63] Ducker G.S., Rabinowitz J.D. (2017). One-Carbon Metabolism in Health and Disease. Cell Metab..

[64] Chen Q., Kirk K., Shurubor Y.I., Zhao D., Arreguin A.J., Shahi I., Valsecchi F., Primiano G., Calder E.L., Carelli V. (2018). Rewiring of glutamine metabolism is a bioenergetic adaptation of human cells with mitochondrial DNA mutations. Cell Metab..

[65] Son S.M., Park S.J., Stamatakou E., Vicinanza M., Menzies F.M., Rubinsztein D.C. (2020). Leucine regulates autophagy via acetylation of the mTORC1 component raptor. Nat. Commun..

[66] Phang J.M. (2019). Proline Metabolism in Cell Regulation and Cancer Biology: Recent Advances and Hypotheses. Antioxid. Redox Signal..

[67] Brooks G.A. (2020). Lactate as a fulcrum of metabolism. Redox Biol..

[68] Passarella S., de Bari L., Valenti D., Pizzuto R., Paventi G., Atlante A. (2008). Mitochondria and L-lactate metabolism. FEBS Lett..

[69] Young A., Oldford C., Mailloux R.J. (2020). Lactate dehydrogenase supports lactate oxidation in mitochondria isolated from different mouse tissues. Redox Biol..

[70] Karamanlidis G., Lee C.F., Garcia-Menendez L., Kolwicz S.C., Suthammarak W., Gong G., Sedensky M.M., Morgan P.G., Wang W., Tian R. (2013). Mitochondrial complex I deficiency increases protein acetylation and accelerates heart failure. Cell Metab..

[71] Schlattner U., Tokarska-Schlattner M., Wallimann T. (2006). Mitochondrial creatine kinase in human health and disease. Biochim. Biophys. Acta.

[72] Schwörer S., Berisa M., Violante S., Qin W., Zhu J., Hendrickson R.C., Cross J.R., Thompson C.B. (2020). Proline biosynthesis is a vent for TGFβ-induced mitochondrial redox stress. EMBO J..

[73] Sarabhai T., Roden M. (2019). Hungry for your alanine: When liver depends on muscle proteolysis. J. Clin. Investig..

[74] Holeček M. (2020). Histidine in Health and Disease: Metabolism, Physiological Importance, and Use as a Supplement. Nutrients.

[75] Cui P., Shao W., Huang C., Wu C.-J., Jiang B., Lin D. (2019). Metabolic derangements of skeletal muscle from a murine model of glioma cachexia. Skelet. Muscle.

[76] Ross-Inta C., Tsai C.Y., Giulivi C. (2008). The mitochondrial pool of free amino acids reflects the composition of mitochondrial DNA-encoded proteins: Indication of a post- translational quality control for protein synthesis. Biosci. Rep..

[77] Mathuthu E., Janse van Rensburg A., Du Plessis D., Mason S. (2021). EDTA as a chelating agent in quantitative 1H-NMR of biologically important ions. Biochem. Cell Biol..

[78] Hafer E., Holzgrabe U., Kraus K., Adams K., Hook J.M., Diehl B. (2020). Qualitative and quantitative ^1^H NMR spectroscopy for determination of divalent metal cation concentration in model salt solutions, food supplements, and pharmaceutical products by using EDTA as chelating agent. Magn. Reson. Chem..

[79] Hornig-Do H.T., Gunther G., Bust M., Lehnartz P., Bosio A., Wiesner R.J. (2009). Isolation of functional pure mitochondria by superparamagnetic microbeads. Anal. Biochem..

[80] Koshenov Z., Oflaz F.E., Hirtl M., Pilic J., Bachkoenig O.A., Gottschalk B., Madreiter-Sokolowski C.T., Rost R., Malli R., Graier W.F. (2021). Sigma-1 Receptor Promotes Mitochondrial Bioenergetics by Orchestrating ER Ca^2+^ Leak during Early ER Stress. Metabolites.

[81] Barbagallo M., Veronese N., Dominguez L.J. (2021). Magnesium in Aging, Health and Diseases. Nutrients.

[82] Huskisson E., Maggini S., Ruf M. (2007). The role of vitamins and minerals in energy metabolism and well-being. J. Int. Med. Res..

[83] Heidari R., Ahmadi A., Mohammadi H., Ommati M.M., Azarpira N., Niknahad H. (2018). Mitochondrial dysfunction and oxidative stress are involved in the mechanism of methotrexate-induced renal injury and electrolytes imbalance. Biomed. Pharmacother..

[84] Alam M.T., Manjeri G.R., Rodenburg R.J., Smeitink J.A., Notebaart R.A., Huynen M., Willems P.H., Koopman W.J. (2015). Skeletal muscle mitochondria of NDUFS4^−/−^ mice display normal maximal pyruvate oxidation and ATP production. Biochim. Biophys. Acta.

[85] Bonilla D.A., Kreider R.B., Stout J.R., Forero D.A., Kerksick C.M., Roberts M.D., Rawson E.S. (2021). Metabolic Basis of Creatine in Health and Disease: A Bioinformatics-Assisted Review. Nutrients.

[86] Lewis C.A., Parker S.J., Fiske B.P., McCloskey D., Gui D.Y., Green C.R., Vokes N.I., Feist A.M., Vander Heiden M.G., Metallo C.M. (2014). Tracing compartmentalized NADPH metabolism in the cytosol and mitochondria of mammalian cells. Mol. Cell.

[87] Szibor M., Gizatullina Z., Gainutdinov T., Endres T., Debska-Vielhaber G., Kunz M., Karavasili N., Hallmann K., Schreiber F., Bamberger A. (2020). Cytosolic, but not matrix, calcium is essential for adjustment of mitochondrial pyruvate supply. J. Biol. Chem..

[88] Yudkoff M. (2017). Interactions in the Metabolism of Glutamate and the Branched-Chain Amino Acids and Ketoacids in the CNS. Neurochem. Res..

[89] Neurauter G., Schrocksnadel K., Scholl-Burgi S., Sperner-Unterweger B., Schubert C., Ledochowski M., Fuchs D. (2008). Chronic immune stimulation correlates with reduced phenylalanine turnover. Curr. Drug Metab..

[90] Nikkanen J., Forsstrom S., Euro L., Paetau I., Kohnz R.A., Wang L., Chilov D., Viinamaki J., Roivainen A., Marjamaki P. (2016). Mitochondrial DNA Replication Defects Disturb Cellular dNTP Pools and Remodel One-Carbon Metabolism. Cell Metab..

[91] Hubbard W.B., Harwood C.L., Prajapati P., Springer J.E., Saatman K.E., Sullivan P.G. (2019). Fractionated mitochondrial magnetic separation for isolation of synaptic mitochondria from brain tissue. Sci. Rep..

[92] McLaughlin K.L., Hagen J.T., Coalson H.S., Nelson M.A.M., Kew K.A., Wooten A.R., Fisher-Wellman K.H. (2020). Novel approach to quantify mitochondrial content and intrinsic bioenergetic efficiency across organs. Sci. Rep..

[93] Ruoppolo M., Caterino M., Albano L., Pecce R., Di Girolamo M.G., Crisci D., Costanzo M., Milella L., Franconi F., Campesi I. (2018). Targeted metabolomic profiling in rat tissues reveals sex differences. Sci. Rep..

[94] Wells A., Barrington W., Threadgill D., Dearth S., Campagna S., Saxton A., Voy B. (2016). Gene, Sex and Diet Interact to Control the Tissue Metabolome. FASEB J..

[95] Miller H.C., Louw R., Mereis M., Venter G., Boshoff J.-D., Mienie L., Van Reenen M., Venter M., Lindeque J.Z., Domínguez-Martínez A. (2021). Metallothionein 1 overexpression does not protect against mitochondrial disease pathology in *Ndufs4* knockout mice. Mol. Neurobiol..

[96] Dona A.C., Kyriakides M., Scott F., Shephard E.A., Varshavi D., Veselkov K., Everett J.R. (2016). A guide to the identification of metabolites in NMR-based metabonomics/metabolomics experiments. Comput. Struct. Biotechnol. J..

[97] Pang Z., Chong J., Zhou G., de Lima Morais D.A., Chang L., Barrette M., Gauthier C., Jacques P.-É., Li S., Xia J. (2021). MetaboAnalyst 5.0: Narrowing the gap between raw spectra and functional insights. Nucleic Acids Res..

