# Supplementary material for: Sub-Cellular Metabolomics Contributes Mitochondria-Specific Metabolic Insights to a Mouse Model of Leigh Syndrome
Source: Metabolites. 2021 Sep 28;11(10):658. doi: 10.3390/metabo11100658 (PMC8537744; doi:10.3390/metabo11100658)
Supplement: Supplementary file 1 [file metabolites-11-00658-s001.zip › metabolites-1386515-supplementary.pdf]

Sub-cellular metabolomics contributes mitochondria-specific metabolic insights to a mouse model of Leigh Syndrome

Gunter van der Walt[1], Jeremie Z. Lindeque[1], Shayne Mason[1] and Roan Louw[1,*]

[1] Human Metabolomics, Faculty of Natural and Agricultural Sciences, North-West University (Potchefstroom Campus), South Africa.
* Correspondence: roan.louw@nwu.ac.za

Supplimentary information

Experimental, data quality & results supplements

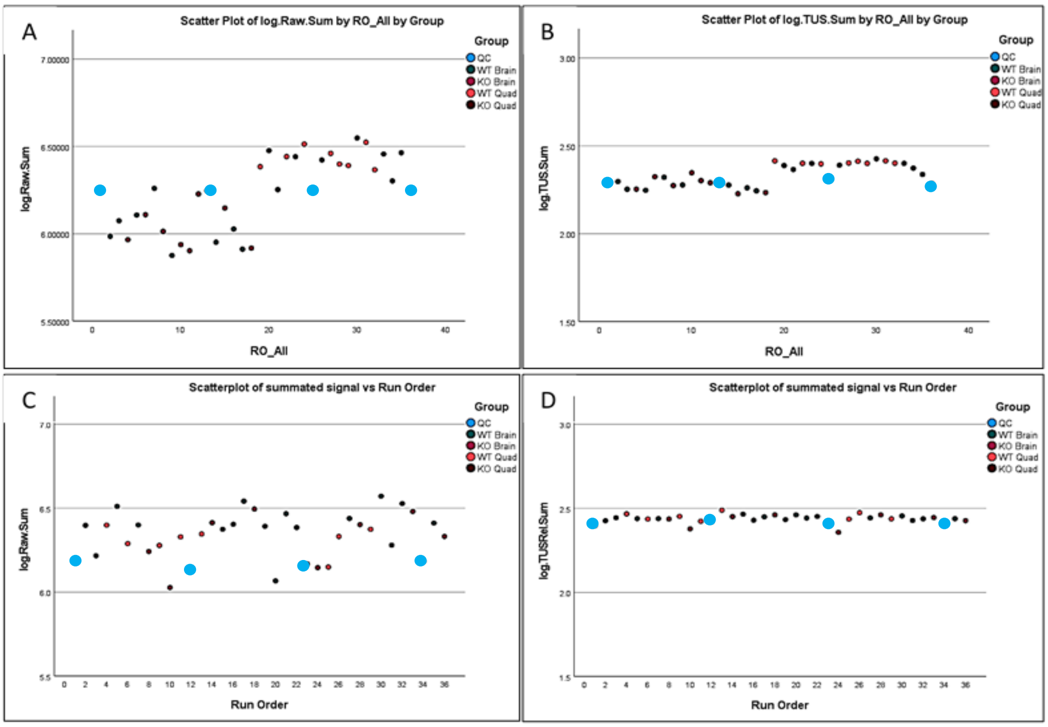

Figure S1: Scatterplots of raw and TUS normalized total feature sum vs run order per NMR batch, indicating applicability of the employed normalization strategy. (A) and **(B)** depict the total sample signal for the unnormalized and TUS normalized cytosol samples, respectively. **(C)** and **(D)** illustrates the same unnormalized and TUS normalized sample sums, respectively for the mitochondrial sample batches. Large blue dots indicate QC replicates throughout each batch.

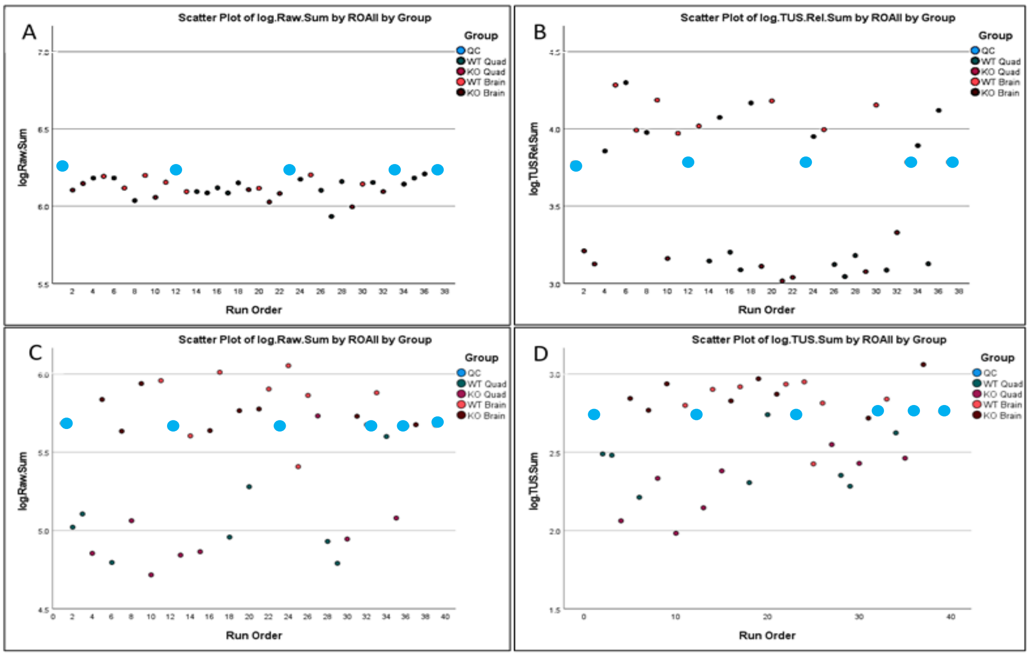

Figure S2: Scatterplots of raw and TUS normalized total feature sum vs run order per LC-MS/MS batch, indicating applicability of the employed normalization strategy. (A) and **(B)** depict the total sample signal for the unnormalized and TUS normalized cytosol samples, respectively. **(C)** and **(D)** illustrates the same unnormalized and TUS normalized sample sums, respectively for the mitochondrial sample batches. Large blue dots indicate QC replicates throughout each batch.

Table S1: **Positively detected features by LC-MS/MS and ^{1}H-NMR**, sorted from highest to lowest detected area signal (unprocessed) Measurement stability is indicated by %CV values for each compound in the separate batches- where N.A. indicates compounds not present above noise level in the specified QC samples.

Metabolite (Level 1)	Med Signal (log mAu)	%QC-CV Mito	%QC-CV Cyto
**LC-MS/MS**			
Carnitine (C0)	5,34	N.A.	3,27
Glutamic acid	5,15	1,48	3,33
Creatine	5,15	N.A.	4,63
Aspartic acid	4,94	2,27	3,72
N-Acetylaspartic acid	4,74	3,49	7,99
γ-Aminobutyric acid	4,65	6,85	3,22
Sarcosine	4,21	N.A.	10,23
Alanine	4,11	3,23	7,73
Serine	4,07	1,02	3,13
Leucine	4,03	1,79	2,59
Glycine	3,96	3,50	9,11
Phenylalanine	3,90	2,41	5,02
Octanoyl-carnitine (C8)	3,86	5,91	38,39
Glutamine	3,87	7,49	4,65
Proline	3,76	12,98	6,50

Metabolite (Level 1)	Med Signal (log mAu)	%QC-CV Mito	%QC-CV Cyto
Valine	3,69	1,23	1,89
Tyrosine	3,63	2,81	3,24
Histidine	3,63	11,06	11,34
Acetylcarnitine (C2)	3,53	2,82	3,11
β-Aminoisobutyric acid	3,75	4,44	5,29
Lysine	3,52	1,94	9,17
Arginine	3,54	5,80	19,39
Isothreo/Threo	3,41	5,34	5,07
Ornithine	3,55	3,67	N.A.
Citrulline	3,33	5,68	17,85
Threonine	3,15	22,30	174,00
Tryptophan	3,09	3,69	N.A.
Methionine	3,11	N.A.	N.A.
Myristoyl-carnitine (C14)	3,04	N.A.	29,81
2-aminoadipic acid	3,05	N.A.	N.A.
4-Hydroxyproline	3,10	N.A.	N.A.
Trimethylglycine	2,99	N.A.	N.A.
**1H-NMR**			
Acetic acid	5,69	11,13	13,82
Formic acid	5,57	27,28	N.A.
Lactic acid	5,56	1,82	3,53
Taurine	5,51	8,78	7,93
Ca(II)-EDTA	5,45	8,06	3,22
Creatine	5,32	3,00	1,19
Mg(II)-EDTA	5,13	9,02	2,58
Carnitine	4,84	8,44	N.A.
Alanine	4,69	N.A.	50,04
Adenosine monophosphate	4,64	N.A.	20,92
N-acetylglutamine	4,52	8,48	10,51
N-acetylaspartic acid	4,51	18,01	4,08
Choline	4,37	13,07	N.A.
Pyruvic acid	4,04	N.A.	31,69

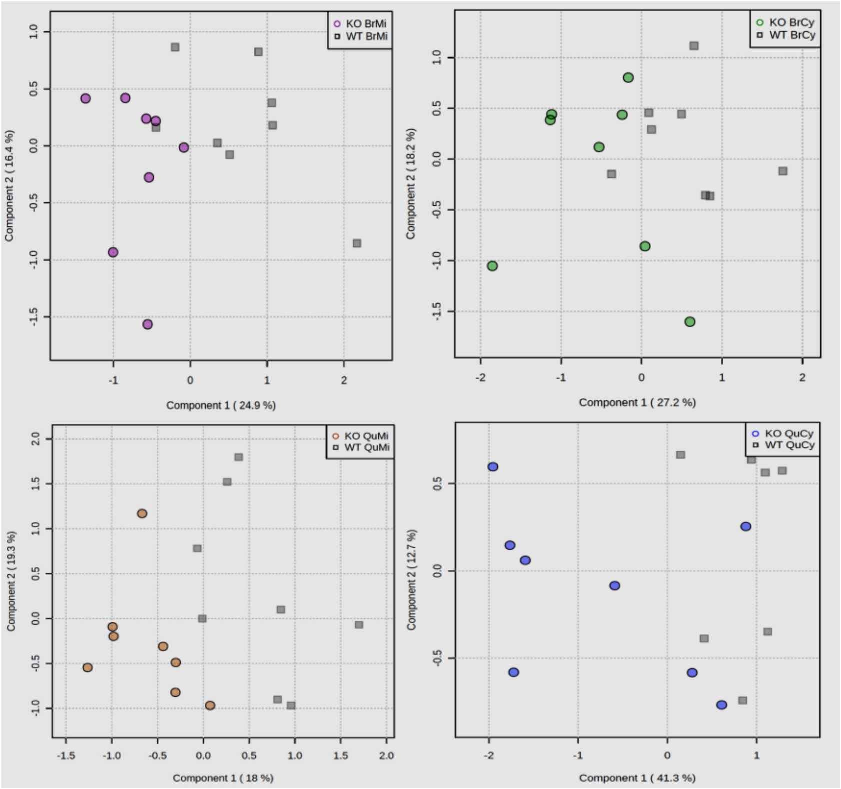

Figure S3. PLS-DA projections of the univariately selected VIP features for each tissue or cell-compartment sample matrix. Figures (A) and (B) represent the PLS model for Ndufs4 KO (color dots) and WT (grey squares) supervised group separation in whole-brain mitochondria and cytosol VIP datasets, respectively. Similarly, (C) and (D) indicate the PLS plots for quadriceps mitochondrial and cytosolic VIP plots, respectively.

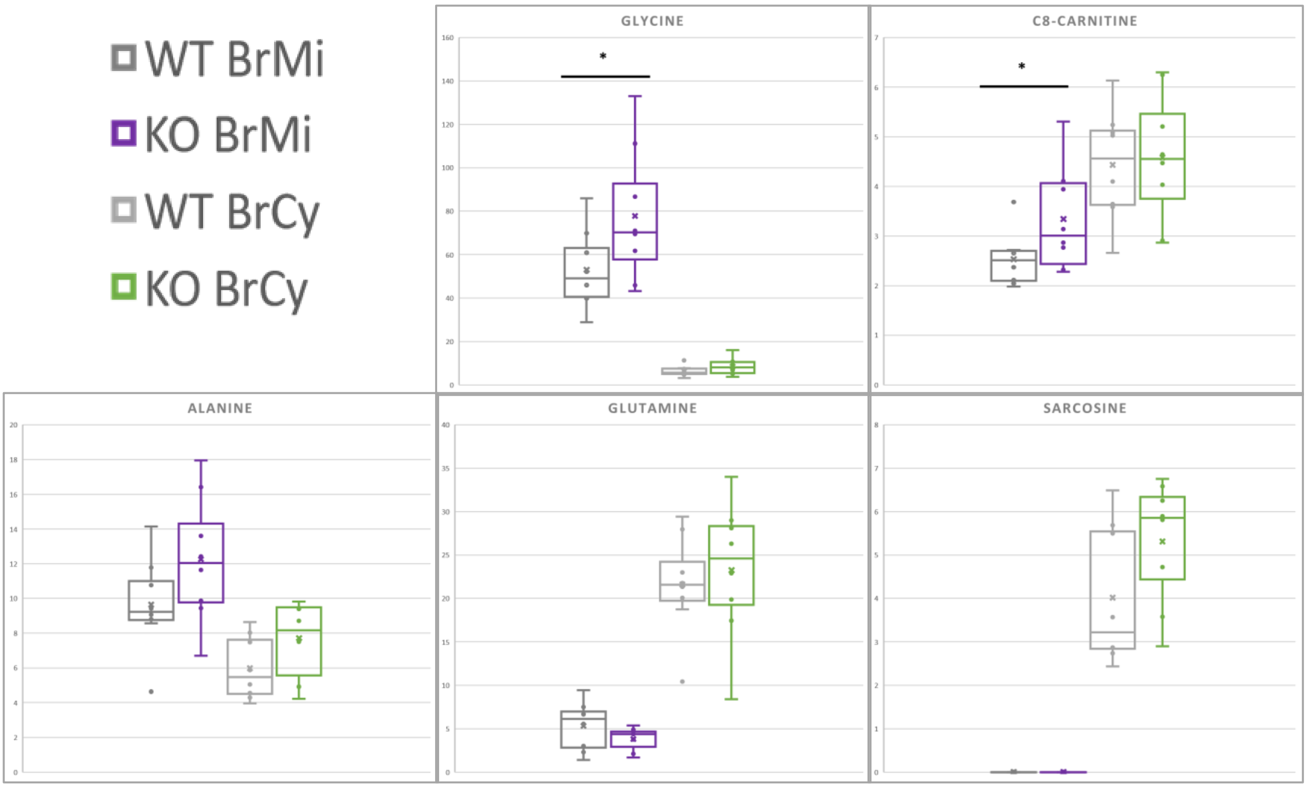

Figure S4. Additional compartment-specific metabolite alterations in Ndufs4 KO whole-brain, identified by ^{1}H-NMR and LC-MS/MS. Boxplots indicate normalized value range descriptives across all analyzed whole-brain matrices. Statistical significance as t-test probabilities is indicated as **(*)** for $p < 0.1$ or (******) for $p < 0.05$.

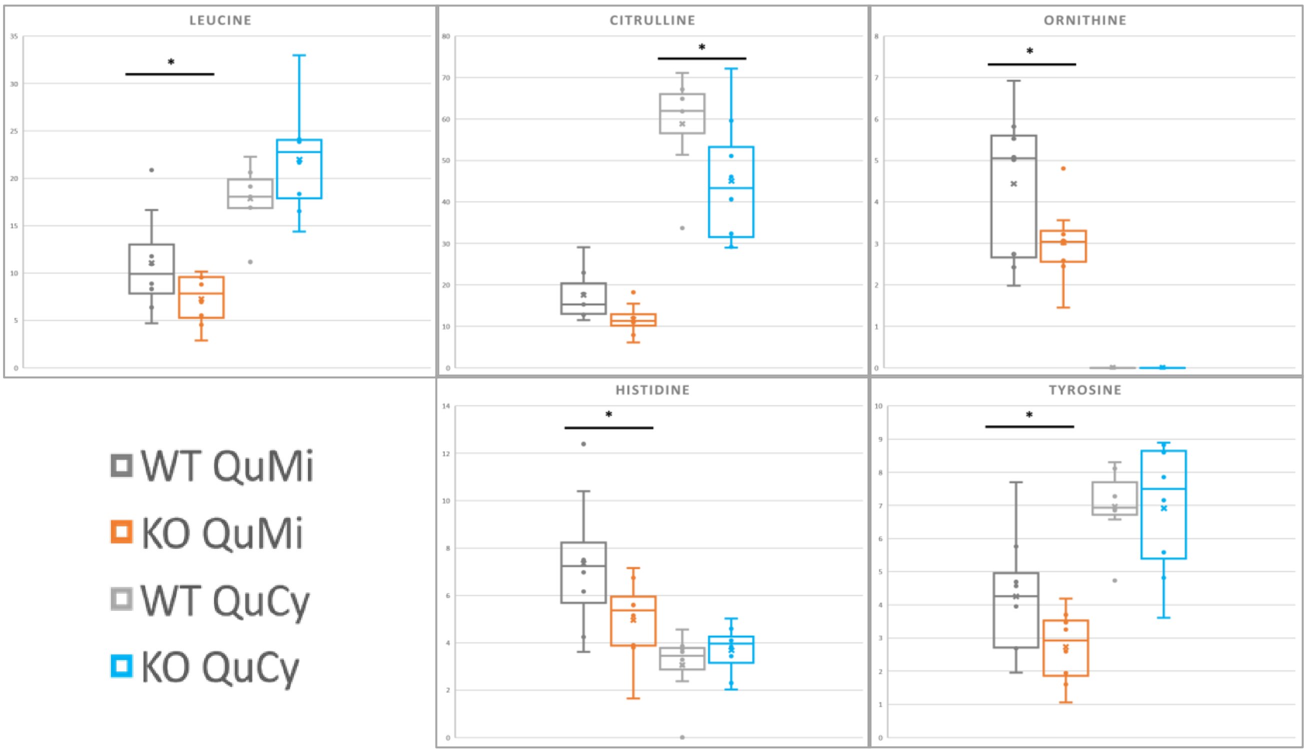

Figure S5. Additional compartment-specific metabolite alterations in Ndufs4 KO quadriceps muscle, identified by ^{1}H-NMR and LC-MS/MS. Boxplots indicate normalized value range descriptives across all analyzed quadriceps matrices. Statistical significance as t-test probabilities is indicated as (*) for $p < 0.1$ or (**) for $p < 0.05$.
